# Treatment Adherence as Predictor of Outcome in Concentrated Exposure Treatment for Obsessive-Compulsive Disorder

**DOI:** 10.3389/fpsyt.2021.667167

**Published:** 2021-06-24

**Authors:** Kristian Tjelle, Håvard Berg Opstad, Stian Solem, Gunvor Launes, Bjarne Hansen, Gerd Kvale, Kristen Hagen

**Affiliations:** ^1^Department of Psychiatry, Møre og Romsdal Hospital Trust, Molde Hospital, Molde, Norway; ^2^Bergen Center for Brain Plasticity, Division of Psychiatry, Haukeland University Hospital, Bergen, Norway; ^3^Department of Psychology, Norwegian University of Science and Technology, Trondheim, Norway; ^4^Division of Psychiatry, Sørlandet Hospital, Kristiansand, Norway; ^5^Department of Clinical Psychology, University of Bergen, Bergen, Norway; ^6^Department of Psychosocial Sciences, University of Bergen, Bergen, Norway; ^7^Department of Mental Health, Norwegian University of Science and Technology, Trondheim, Norway

**Keywords:** obsessive-compulsive disorder, treatment adherence and outcome, exposure and response prevention, concentrated treatment, the Patient EX/RP Adherence Scale, Work and Social Adjustment Scale, quality of life

## Abstract

**Background:** The treatment of choice for obsessive-compulsive disorder (OCD) is exposure and response prevention (EX/RP). Previous studies have demonstrated that treatment adherence predicts treatment outcome for patients with OCD, but there is little knowledge on its role in concentrated exposure treatment for OCD.

**Method:** In the present study, 42 patients received EX/RP treatment using the Bergen 4-day format. Adherence was measured with the Exposure and Response Prevention Adherence Scale (PEAS, rated both by patients and therapists) after the second and third day. Treatment outcome (symptoms of OCD, depression, anxiety, work- and social functioning, and well-being) was assessed at 3-month follow-up.

**Results:** At follow-up, 71.4% were in remission. High adherence was reported (mean score of 6 on a 1–7 scale). The combination of patient- and therapist rated adherence was significantly associated with treatment outcome whilst controlling for age, sex, and pre-treatment scores. Patients with higher degree of adherence reported less symptoms, higher functioning, and more well-being at follow-up.

**Conclusions:** The results of the present study indicated that adherence in concentrated exposure treatment is significantly associated with a wide range of treatment outcomes for OCD.

## Introduction

Exposure and response prevention (EX/RP) is an effective treatment for obsessive-compulsive disorder (OCD), and is recommended in treatment guidelines (1). EX/RP is effective in various formats (2–4), including brief, concentrated, or intensive treatment (5–8). Intensive treatments have been defined as interventions lasting <4 weeks, and often involves daily sessions (9). Intensive formats have shown similar effects as more standard treatments which use weekly or twice-weekly sessions (5, 7, 10). The Bergen 4-day format (B4DT) is one example of such concentrated treatment for OCD. Patients receive individual treatment in a group setting. The B4DT demonstrated promising treatment outcomes (11–15). Studies investigating the long-term outcome, report a recovery rate around 70% at 1- and 4-year follow-up (16, 17).

Some early studies suggested that adherence to EX/RP procedures was associated with positive treatment outcomes (18–23), while others found non-significant results [e.g., (24)]. These studies had different ways of assessing adherence including measures without established reliability and validity. Some of the studies also suggested that certain types of adherence could be more important than others. For instance, one of the studies found that understanding the treatment rationale and compliance with in-session and homework exposure instructions were related to outcome (19). However, they did not find ritual prevention and self-monitoring of rituals to be important.

To address issues concerning multiple different ways of assessing adherence, the Patient EX/RP Adherence Scale (PEAS) was developed (25). The PEAS quantifies how well patients adhere to exposure tasks. The authors devised the PEAS to tap components of standard EX/RP thought necessary for good outcomes (26). These involve confronting fears and stopping rituals (27). The PEAS included three items: (1) the number of exposures the patient attempted (as a percentage of those assigned), (2) the quality of attempted exposures, and (3) the patient's degree of success with response prevention. As a global measure of patient adherence, the three PEAS items are averaged at each session and then across all sessions.

Some studies have investigated treatment adherence in EX/RP for OCD using the PEAS, but there is no knowledge about the role of patient adherence in concentrated treatment formats. For standard OCD treatment, Simpson et al. (21, 22) found that higher scores on the PEAS predicted lower OCD severity at post-treatment and 6-month follow-up in a sample of 30 patients receiving twice-weekly EX/RP. Similarly, Wheaton et al. (23) found that therapist rated adherence strongly predicted symptom severity at post-treatment for twice-weekly EX/RP. More specifically, they found that it was especially the third component of the PEAS (being successful with the exposure assignments) that explained most variance in treatment outcome. They also found that response prevention tended to increase across sessions, and that patients doing response prevention 90% of the time were successful in treatment, while patients with 75% or less had poor prognosis. Patients' adherence with EX/RP homework also predicted post-treatment outcome (but not follow-up) in a sample of 50 patients with OCD receiving twice-weekly EX/RP (28).

Traditionally, studies on the effect of treatment adherence have focused on symptom severity rather than patients' functioning. Although, EX/RP is found to be effective for symptom severity, less is known about its impact on functional impairment (29–31) and well-being (e.g., positive mental health). Functional impairment refers to difficulties with engaging in daily-life activities such as work and socially, due to psychological symptomatology (32). To investigate functional impairment it is recommended to make use of self-report instruments to take into account patients' own subjective experiences (33, 34). To our knowledge, there have been no studies relating treatment adherence to functional impairment like work- and social adjustment and well-being. It is also the first of its kind to do so under the investigation of a brief, concentrated, or intensive treatment form.

The aim of the present study was therefore to explore whether patients' adherence to treatment principles of the concentrated EX/RP-treatment predict treatment outcome at post-treatment and 3-month follow-up in a concentrated EX/ER format. We hypothesized that high adherence to treatment principles would be associated with better treatment outcomes. We also expected that adherence scores would be higher than in standard EX/RP given the brief time interval and the close contact with therapist. Furthermore, we wanted to investigate whether these ratings also predicted changes within the domains of well-being and functional impairment. The main hypothesis was that treatment adherence would be associated with better treatment outcomes across all measures.

## Methods

### Participants and Procedure

Treatment was delivered as part of the specialist health care service in Norway. The study was part of a randomized controlled trial completed at Sørlandet Hospital. The study was approved by the Regional Committee for Medical and Health Research Ethics (Reference Number 2016/794) in addition to being registered in ClinicalTrials.gov (Identifier: NCT02886780). All patients signed an informed consent before inclusion to the study.

Eligible patients for OCD treatment were referred from their general practitioner to the OCD-team, which is part of the public specialist outpatient mental health care. Patients referred to the clinic were offered the opportunity to either opt for the concentrated treatment study, or the standard treatment offered at the clinic (individual EX/RP with weekly sessions). They were then randomized either to B4DT (*n* = 16), a 3-month unguided self-help (SH; *n* = 16) based on a manual by Kozak and Foa (26), or a 3-month waiting list (WL; *n* = 16). The patients that were randomized to the SH- or WL-condition who wanted more treatment were offered the B4DT after the initial intervention period. In total, 26 of the 32 patients (81.3%) requested to do so. The total sample size for this study was therefore 42. The patients were assessed at pre-treatment, post-treatment, and 3-month follow-up.

Referred patients were screened and evaluated for eligibility using the Structured Clinical Interview for DSM-5 [SCID-5; (35)]. Severity of OCD symptoms was assessed using the Yale-Brown Obsessive Compulsive Scale [Y-BOCS; (36)]. The SCID-5 and Y-BOCS interviews were conducted by an experienced and independent assessor. Criteria for inclusion were as follows: the patient had to be 18 years or older and fluent in Norwegian, fulfilling diagnostic criteria for OCD according to the DSM-5 and have a score on the Y-BOCS of 16 points or more.

Patients with ongoing substance abuse/dependence, bipolar disorder, psychosis, suicidal ideation or plans, and intellectual disability (based on previous medical history) were excluded. Patients were also excluded if antidepressants had not been stabilized or if they were unwilling to refrain from anxiolytics and alcohol during the 2 days of exposure. All patients included complied with the aforementioned pre-requisites. Due to an ongoing national trial for treatment non-responders, patients with a full course of prior CBT for OCD were referred to that study instead.

The sample consisted of 42 patients (76.2% female) with a mean age of 30.1 (SD = 10.7). Demographics and clinical characteristics prior to treatment are displayed in Table 1. The OCD symptom intensity for the group as a whole was moderate to severe. In addition, the sample showed moderate symptoms of depression and generalized anxiety. Close to half of the sample (45.2%) received some type of disability benefit, 33.3% worked, and 21.4% were students. A total of 45.2% used some type of psychotropic medication (26.2% used SSRIs).

**Table 1:** Sample characteristics and change in symptoms, well-being, and work- and social functioning.

	***N* (%)**		***M* (SD)**
Female sex	32 (76.2)	Treatment adherence	
Civil status		PEAS therapist	6.23 (0.61)
Single	17 (40.5)	PEAS Patient	5.66 (0.68)
Married	11 (26.2)	PEAS combination	5.94 (0.53)
Cohabitant	12 (28.6)	Symptoms	
Separated	2 (4.8)	Y-BOCS	
		Pre	27.60 (3.87)
Work status		Post	11.92 (4.45)
Employed	14 (33.3)	Follow-up	9.66 (5.99)
Student	9 (21.4)	PHQ-9	
Other	19 (45.2)	Pre	12.83 (5.44)
		Post	8.03 (3.81)
Comorbid disorders	37 (88.1)	Follow-up	7.53 (4.73)
Anxiety	25 (59.5)	GAD-7	
Depression	18 (42.9)	Pre	12.90 (4.40)
		Post	8.12 (3.68)
		Follow-up	6.59 (4.21)
		WSAS	
Using psychotropics	19 (45.2)	Pre	18.24 (7.80)
		Follow-up	9.73 (7.67)
	*M* (SD)	WEMWBS	
Age	30.05 (10.74)	Pre	40.29 (8.14)
Years of school	12.62 (1.91)	Follow-up	44.98 (8.92)

*Psychotropics used = SSRI/SNRI (n = 11), anxiolytic (n = 3), hypnotics (n = 1), anti-psychotics (n = 2), Ritalin (n = 1), anti-epileptics (n = 1). PEAS, The Patient EX/RP Adherence Scale; Y-BOCS, The Yale-Brown Obsessive-Compulsive Scale; PHQ-9, Patient Health Questionnaire-9; GAD-7, Generalized Anxiety Disorder Scale-7; WSAS, The Work and Social Adjustment Scale; WEMWBS, The Warwick-Edinburgh Mental Wellbeing Scale*.

#### Treatment

All patients received the Bergen 4-day treatment (B4DT), which is a concentrated EX/RP treatment delivered during 4 consecutive days (16, 17). The treatment was delivered in a combination of a group setting and individual EX/RP, delivered simultaneously to 3–6 patients by the same number of therapists.

The first day (3 h) consisted of psychoeducation of EX/RP in a group setting and preparation of individual tailored exposure tasks for the coming days. The second day starts with a demonstration on how to maximize the effect of EX/RP. This demonstration is carried out both in the group setting and individually. The patients are encouraged to do the exposure without any subtle avoidance and refrain from all safety behavior, which is explained as “lean into the anxiety” [see (16)]. For the remainder of the second day and the third day (8 h each day), patients were engaged in therapist-assisted EX/RP conducted in a wide range of settings (primarily outside the clinic). In the afternoon, the patients were encouraged to continued self-administered EX/RP and report to their therapist on their progress. In the afternoon of the third day, the patients' friends and relatives were invited to a psychoeducation meeting (1.5 h). The fourth day starts with a summary of the treatment, planning how to continue EX/RP on their own, and focus on relapse prevention. Three months after the treatment, the patients were scheduled for a follow-up session (30 min), with focus on repetition of the treatment components [see (13) for further description of the treatment].

#### Measures

*The Patient EX/RP Adherence Scale [PEAS;* *(**25**)]* is a 3-item form, which assesses the patient's between-session adherence to the therapist's EX/RP instructions. The scale was designed to focus on the key procedures of EX/RP and to be brief enough to be used after each treatment session. The scale demonstrated excellent inter-rater reliability and good face- and content validity (25). Assessments of adherence were carried out both by therapist and the patients themselves at the end of the 2 days of exposure (day 2 and 3). Both therapist- and patient rated PEAS were scored as an overall impression of within-session adherence (therapist-assisted exposure and partly-therapist assisted exposure) and between-session adherence (unassisted homework assignments in the afternoon). The therapists' adherence ratings were scored before treatment the following day, while the patients' adherence ratings were scored either in the afternoon (late afternoon) or the next morning. Combined scores of the PEAS were calculated by averaging the patient- and therapist rated scores (i.e., a patient rated score of 6.5 and a therapist rated score of 5.5 equaled to a combined score of 6.0).

The first item of the PEAS concerns percentage of exposures that the patient attempted. Scores range from 1 (none, 0%) to 7 (all, 100%). A score of 4 equals to 50%. The second item concerns how well the patient did the assigned exposures. Scores range from 1 (refused, none) to 7 (excellent, all of the exposures attempted were performed as assigned by the therapist). A score of 4 equals to making a good effort to conduct the exposures but giving into compulsions during or after the exposure. The third and final item concerns response prevention (e.g., to what extent the patient successfully resisted the urge to ritualize). Scores range from 1 (none) to 7 (most, above 90%). A score of 4 on item 3 equals to 50%. For the current study, we used mean item scores when reporting PEAS results.

*The Yale-Brown Obsessive-Compulsive Scale [Y-BOCS;* *(**36*, *37**)]* was used to assess severity of OCD symptoms. The scale consists of a symptom checklist covering obsessions and compulsions and a severity scale. The severity scale consists of different 10 items, rated on a 5-point scale ranging from 0 (no symptoms) to 4 (severe symptoms). The total score ranges from 0 to 40.

*The Patient Health Questionnaire-9 [PHQ-9;* *(**38**)]* is a 9-item self-administered screening instrument for depression. The total score ranges from 0 to 27. A score of 10 or more is considered indicative of a depressive disorder. The psychometric properties of PHQ-9 are well-established (39, 40).

*Generalized Anxiety Disorder Scale [GAD-7;* *(**41**)]* is a 7-item measure of generalized anxiety symptoms. The total score ranges from 0 to 21. The psychometric properties are well-established (38, 42).

*The Work and Social Adjustment Scale [WSAS;* *(**29**)]* is a five-item questionnaire that focus on an individual's impairment in areas of work, social and private activities, functioning at home and close relationships. Each item is rated on a 9-point scale ranging from 0 (not at all) to 8 (very severe). Total scores range from 0 to 40, with higher scores indicating higher levels of functioning impairment. The WSAS has good internal consistency and test–retest reliability (29, 43).

The Warwick-Edinburgh Mental Well-being Scale [WEMWBS; (44)] is a 14-items questionnaire covering issues such as positive affect, level of functioning, and relationships over the past 2 weeks. Total scores range from 14 to 70 with higher scores indicating greater well-being. The WEMWBS scale has good psychometric properties (45).

### Statistical Analyses

To investigate the relationship between adherence and OCD symptoms, we used Pearson correlations. We also conducted five hierarchical multiple regression analyses to examine treatment adherence as a predictor of OCD symptoms, symptoms of anxiety and depression, well-being, and work- and social function at 3-month follow-up. The regressions controlled for age and sex (step 1), and the pre-treatment value of the dependent variable (step 2). The treatment adherence scores was computed by combining the patient- and therapist rated versions of PEAS (step 3).

Missing data were imputed using expectation maximization (EM). The dataset had a relatively low amount of missing data (3.3%). For imputing the missing data, outcome variables at each time point were included (46). The missing data were found to be completely at random [Little's MCAR test $\chi_{\left(523\right)}^{2}$ = 500.03, *p* = 0.758].

## Results

Treatment was associated with improvement in OCD symptoms. At follow-up, 71.4% (*n* = 30) were classified as in remission (scoring 12 or below on Y-BOCS and having at least 35% improvement on Y-BOCS). The within-group effect size (using pooled SD) from pre-treatment to follow-up was 3.56 for Y-BOCS. For the other outcomes measures the effect sizes were 1.47 (GAD-7), 1.04 (PHQ-9), 1.10 (WSAS), and −0.55 (WEMWBS).

Both patients and therapists rated strong adherence (mean score of 6 on a 1–7 scale). Therapists rated adherence slighter higher than patients (see Table 1). This difference equaled to an effect size of 0.88. Patients' ratings for the three items of PEAS were quite similar with a mean of 5.9 (SD = 0.9) for item 1 (doing all the exposures), 5.4 (SD = 0.8) for item 2 (quality of exposures), and 5.6 (SD = 0.9) for item 3 (response prevention).

Treatment adherence (patient- and therapist rated) was significantly correlated with Y-BOCS scores at post-treatment (*r* = −0.59, *p* < 0.001) and 3-month follow-up (*r* = −0.54, *p* < 0.001). In general, the combined scores showed stronger correlations with treatment outcome, than therapist- or patient rated adherence alone. Treatment adherence (combined variable) was significantly correlated with all outcome measures (except GAD-7 post-treatment). There were no significant correlation between pre-scores for any of the outcome measures and adherence with the exception of Y-BOCS and WEMWBS. Higher Y-BOCS scores at pre-treatment were associated with lower patient rated adherence. Higher WEMWBS scores were associated with higher patient rated adherence. See Table 2 for further details.

**Table 2:** Relationship between treatment adherence and treatment outcome measures.

	**PEAS**		
	**Therapist rated**	**Patient rated**	**Combination**
**Y-BOCS**			
Pre	0.10	−0.34^*^	−0.16
Post	−0.55^**^	−0.42^**^	−0.59^**^
Follow-up	−0.42^**^	−0.45^**^	−0.54^**^
**PHQ-9**			
Pre	−0.06	−0.33^*^	−0.25
Post	−0.37^*^	−0.27	−0.39^*^
Follow-up	−0.44^**^	−0.42^**^	−0.53^**^
**GAD-7**			
Pre	0.21	−0.04	0.10
Post	−0.16	−0.23	−0.24
Follow-up	−0.36	−0.41^*^	−0.47^**^
**WSAS**			
Pre	0.33	−0.17	0.09
Follow-up	−0.35^*^	−0.37^*^	−0.44^**^
**WEMWBS**			
Pre	0.10	0.35^*^	0.28
Follow-up	0.42^**^	0.33^*^	0.46^**^

*PEAS, Patient Exposure/Response Prevention Adherence Scale; Y-BOCS, Yale-Brown Obsessive Compulsive Scale; PHQ-9, Patient Health Questionnaire 9; GAD-7, Generalized Anxiety Disorder 7; WSAS, Work and Social Adjustment Scale; WEMWBS, The Warwick-Edinburgh Mental Wellbeing Scale.*

* *p < 0.05*,

** *p < 0.01*.

Five hierarchical multiple regression analysis were used to assess the ability of the combined therapist and patient rated PEAS to predict 3-month follow-up scores for all outcome measures. PEAS was a significant predictor for Y-BOCS, PHQ-9, GAD-7, WSAS, and WEMWBS. Age and sex on step 1 was not significant for any of the five regressions. A summary of the regression analyses is displayed in Table 3. For Y-BOCS, the *R*^2^ was 0.29 (Adj. *R*^2^ = 0.211). Pre-treatment Y-BOCS on step 2 was not significant. However, PEAS on step 3 was significant, explaining an additional 25.6% of the variance.

**Table 3:** Treatment adherence as a predictor of 3-month follow-up.

	**Y-BOCS**			**PHQ-9**			**GAD-7**			**WSAS**			**WEMWBS**		
	**Adj. *R*^**2**^**	***F* cha**	**Sign *F* cha**	**Adj. *R*^**2**^**	***F* cha**	**Sign *F* cha**	**Adj. *R*^**2**^**	***F* cha**	**Sign *F* cha**	**Adj. *R*^**2**^**	***F* cha**	**Sign *F* cha**	**Adj. *R*^**2**^**	***F* cha**	**Sign *F* cha**
Age and sex	−0.02	0.56	0.575	−0.02	0.64	0.534	0.02	1.38	0.264	0.00	1.03	0.368	0.05	2.04	0.144
Pre	−0.05	0.14	0.710	0.18	10.16	0.003	0.04	1.92	0.174	0.15	7.61	0.009	0.43	27.21	<0.001
PEAS	0.21	13.31	0.001	0.37	12.75	0.001	0.33	17.69	<0.001	0.31	10.29	0.003	0.52	7.95	0.008
**Final step of the equation**															
	**β**	***t***	***p***	**β**	***t***	***p***	**β**	***t***	***p***	**β**	***T***	***p***	**β**	***t***	***p***
Age	−0.01	−0.05	0.963	−0.14	−1.02	0.315	−0.27	−1.97	0.057	−0.06	−0.46	0.647	0.23	2.04	0.049
Sex	0.04	0.29	0.776	−0.04	−0.29	0.775	−0.08	−0.60	0.551	0.25	1.76	0.088	−0.03	−0.27	0.792
Pre	−0.00	−0.03	0.978	0.34	2.53	0.016	0.26	1.98	0.056	0.42	3.13	0.003	0.54	4.69	<0.001
PEAS	−0.53	−3.65	0.001	−0.48	−3.57	0.001	−0.56	−4.21	<0.001	−0.43	−3.21	0.003	0.33	2.82	0.008

*Pre, pre-treatment value of the dependent variable; PEAS, Patient Exposure/Response Prevention Adherence Scale; Y-BOCS, Yale-Brown Obsessive Compulsive Scale; PHQ-9, Patient Health Questionnaire 9; GAD-7, Generalized Anxiety Disorder 7; WSAS, Work and Social Adjustment Scale; WEMWBS, The Warwick-Edinburgh Mental Wellbeing Scale*.

For PHQ-9, the *R*^2^ was 0.43 (Adj. *R*^2^ = 0.37). Pre-treatment PHQ-9 was significant on step 2 explaining 20.4%. PEAS on step 3 added another 19.6% of explained variance and was a significant predictor. For GAD-7 the *R*^2^ was 0.40 (Adj. *R*^2^ = 0.33). Pre-treatment GAD-7 on step 2 was not significant, but PEAS on step 3 added an additional 28.8% of explained variance.

For WSAS the *R*^2^ was 0.38 (Adj. *R*^2^ = 0.31). Pre-treatment WSAS on step 2 explained 15.9% of the variance. PEAS on step 3 added another 17.2% of explained variance. Finally, for WEMWBS, the *R*^2^ was 0.57 (Adj. *R*^2^ = 0.52). Step 1 was not significant. Pre-treatment WEMWBS explained 37.8%, while PEAS on step 3 added another 9.3% of explained variance.

**Figure 1:**
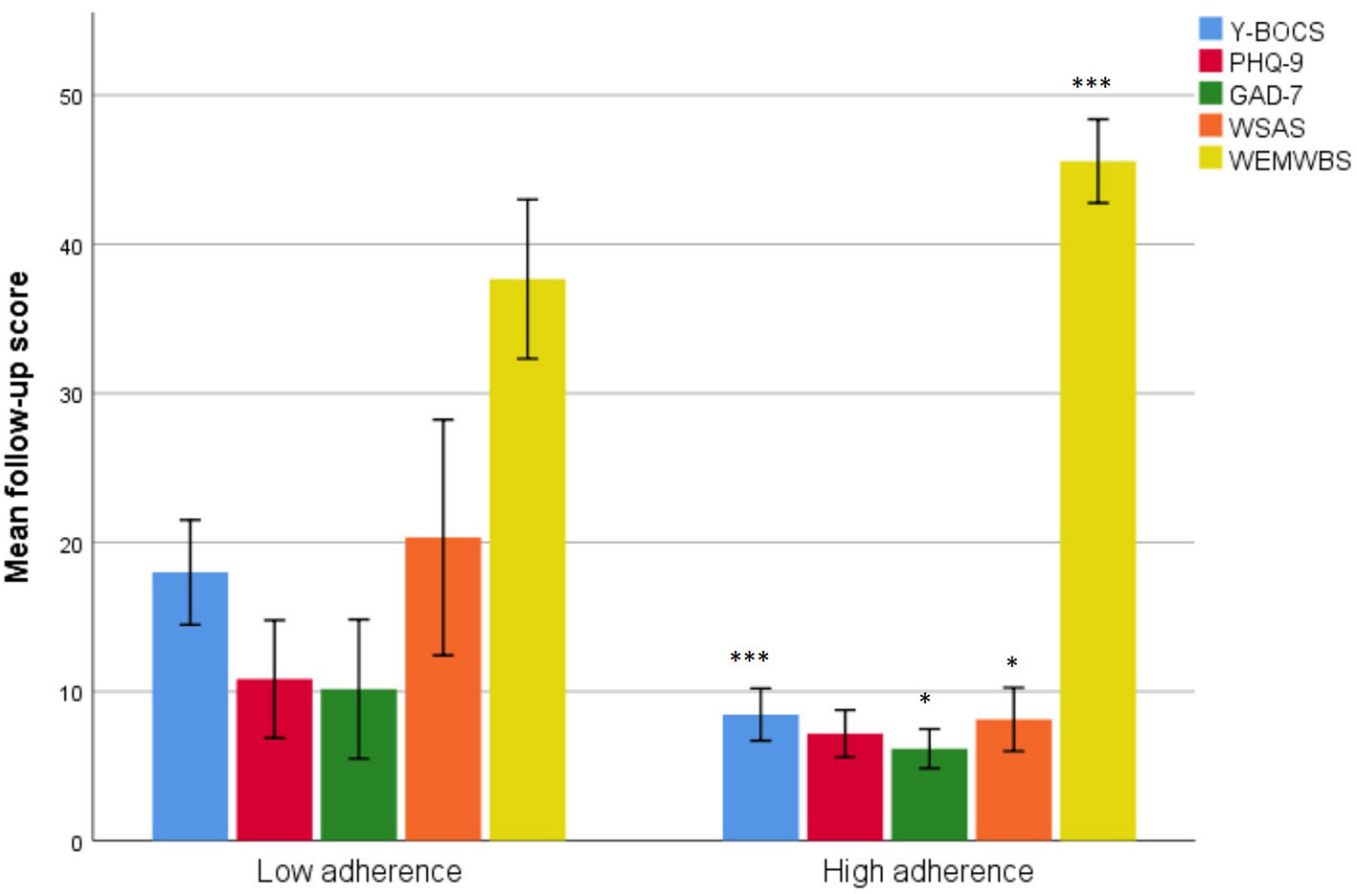
Comparisons between patients rating themselves high or low on quality of exposures attempted. *** < 0.001, * < 0.05.

Comparisons were made between patients' ratings on item 2 (quality of the exposure exercise or how well they did the exposures) of the PEAS. Patients were rated as low on adherence if they had a score below 5 (score of 4 equalled to “made a good effort to conduct the exposures as assigned by the therapist but gave into compulsions during or after the exposure”). Patients scoring 5 or higher [score of 5 equalled to “good, completed the exposures as assigned by the therapist (e.g., appropriate exposure, correct amount of time) with minimal compulsions or safety aids during or afterwards] were rated as high on adherence. Patients scoring themselves low on adherence had more symptoms of OCD and anxiety as well as lower work- and social functioning at 3-month follow-up. They also had lower scores on well-being. A graphical summary is displayed in Figure 1.

## Discussion

This study investigated the relationship between patients' adherence to EX/RP principles and treatment outcome. As predicted, there was a relatively strong relationship between adherence and treatment outcome. The strongest relationships were found when using the combined adherence score (both patient- and therapist rated). The present results are in line with previous studies that have revealed similar patterns in standard OCD treatment (21–23). This thereby strengthens the evidence for adherence as a predictor of treatment outcome. The study also extends these findings, by using a concentrated treatment format, and showing that adherence was also related to symptoms of anxiety and depression, well-being, and work- and social functioning. This implies that adherence could be an important factor for successful treatment of OCD.

The adherence scores were quite high with mean scores of 6.2 (therapist rated) and 5.7 (patient rated) on a 1–7 scale. Also, there were quite small standard deviations (0.6–0.7). These mean scores are considerably higher than previous studies [e.g., (25)]. The discrepancy may be related to differences in measuring treatment adherence. In this study, some of the exposure tasks were therapist-assisted, others partly assisted, whilst homework assignments were unassisted. The therapist's rating was a total impression based on within- and between-session (homework) adherence. The rating summarized adherence to all exposure tasks conducted that day. In comparison, other studies have limited ratings to between-session adherence.

The PEAS is only scored for 2 days of exposure, and it might me easier to do exposure tasks when the time-interval is so brief. Much of the day is spent together with the therapist, and there is also contact between the patient and the therapist in the period when the patient do homework. The treatment also stresses the importance of having a clear plan for homework, which might increase the adherence. Therefore, the intensive format, the close contact with the therapist, and ratings that include both within- and between-session adherence, are all likely reasons for the difference in adherence scores between studies. Other possible reasons for the high adherence scores could be related to the concentrated format making it easier for the patient to adhere to the treatment principles given the short time period and that patients selecting this format are more motivated or able to sustain motivation during this brief period.

The change in OCD symptoms from pre-treatment to follow-up was large (*d* = 3.56), and likely related to the high level of adherence reported. This finding is important for patients who struggle with motivation for EX/RP treatment. The results suggested that the quality of exposure tasks attempted should be rated 5 (“good”) or higher by the patients. Scores below 5 should be taken as indications that therapeutic interventions may be needed in order to strengthen adherence.

The fact that therapists rate patients as more adherent than the patients rated themselves could indicate that patients tend to be more self-critical. But it could also be due to them having more information about exposure and response prevention in situations where the therapists was not present. Another explanation could be that it may be less clear for patients to distinguish rituals and avoidance from normal behavior. Therapist rated adherence was not related to patients' pre-treatment levels of OCD, depression, anxiety, well-being, or functioning. However, there were significant correlations between patients' ratings of adherence and their symptoms (OCD and depression but not anxiety symptoms) and well-being (but not functioning). This could be due to report style or a possible indication that it is more difficult to adhere for patients with higher severity.

A relevant aspect to the role of adherence in treatment of OCD concerns how therapists can increase compliance. One study suggested that the theraputic alliance and motivation was associated with adherence to OCD treatment (47). As discussed by the authors, this suggests that taking time to prepare patients for treatment, collaborativly developing a case formulation, and ensuring that the patient understands and agrees with the treatment rationale before conducting exposure can have a strong impact on adherence and outcome (48–50). This corroborates with related findings suggesting that understanding the treatment rationale and compliance with in-session and homework exposure instructions are related to outcome (19). Future studies could explore if it is possible to manipulate degree of adherence. This could involve adjusting treatment rationale, case formulation, and motivational interventions, but also explore other possible factors associated with adherence.

There is a limitation that the study only included short-term follow-up data and there was no inter-rater reliability statistic. Therefore, it is still unknown whether adherence affects long term treatment outcomes. Also, we do not know how well the patients adhered to treatment principles after the treatment period was over. Furthermore, the sample size limited the number of variables that could be included in the regression analyses. It is also a limitation that the study did not include patients that had been previously treated with EX/RP, because there was an ongoing parallel study for difficult-to-treat OCD-patients (51). Therefore, future studies should investigate the role of adherence using larger samples and in patients that have relapsed or not responded to previous treatments.

It has previously been discussed that it could be conceptually difficult to disentangle treatment compliance from treatment outcome as one would expect considerable overlap (19). It was posited that adherence to exposure instructions is both compliance and progress (outcome) and could thereby explain strong relationships between the two. Especially the third item of PEAS overlaps with Y-BOCS items concerning patients' ability to resist and control compulsions. However, in the current study we also included outcome measures that are not directly connected to OCD. And the results were in similar; adherence was related also to depression, anxiety, well-being, and functioning, not only symptoms of OCD.

In conclusion, the results of the present study indicated that adherence to the treatment was an important factor for treatment outcome. This finding was not restricted to symptoms of OCD. Adherence was also important for symptoms of anxiety and depression, well-being, and work- and social functioning. Future research should explore strategies aimed at improving patient adherence and thereby potentially improve treatment outcome.

## Data Availability Statement

The raw data supporting the conclusions of this article will be made available by the authors, without undue reservation.

## Ethics Statement

The studies involving human participants were reviewed and approved by the Regional Committee for Medical and Health Research Ethics Number 2016/794. Written informed consent to participate in this study was provided by the participants' legal guardian/next of kin.

## Author Contributions

BH, GK, GL, and KH contributed to the study design. KT, SS, and KH contributed to the statistical analysis and drafted the manuscript. All authors contributed to the revision of the manuscript and approved the final version of the manuscript.

## Conflict of Interest

The authors declare that the research was conducted in the absence of any commercial or financial relationships that could be construed as a potential conflict of interest.
